# Impact of a novel protocol for atrial fibrillation management in outpatient gastrointestinal endoscopic procedures: a retrospective cohort study

**DOI:** 10.1186/s12872-018-0915-0

**Published:** 2018-09-03

**Authors:** Joseph Longino, Ashish Chaddha, Matthew M. Kalscheur, Anne M. Rikkers, Deepak V. Gopal, Michael E. Field, Jennifer M. Wright

**Affiliations:** 1 Department of Medicine, Division of Cardiology, University of Wisconsin School of Medicine and Public Health, 600 Highland Avenue, Madison, WI 53792 USA; 2 Department of Cardiology, Beaumont Hospital, Royal Oak, MI USA; 3 Department of Emergency Services, University of Wisconsin School of Medicine and Public Health, Madison, WI USA; 4 Department of Medicine, Division of Gastroenterology & Hepatology, University of Wisconsin School of Medicine and Public Health, Madison, WI USA; 5 Department of Medicine, Division of Cardiology, Medical University of South Carolina and Ralph H. Johnson Veterans Administration Medical Center, 30 Courtenay Drive, Charleston, SC 29425 USA

**Keywords:** Atrial fibrillation, Endoscopy, Emergency medicine

## Abstract

**Background:**

Atrial fibrillation (AF) may result in procedure cancellations and emergency department (ED) referrals for patients presenting for outpatient GI endoscopic procedures. Such cancellations and referrals delay patient care and can lead to inefficient use of resources.

**Methods:**

All consecutive patients presenting in AF for a colonoscopy or upper endoscopy to the University of Wisconsin Digestive Health Center between October 2013 and September 2014 were defined as the pre-intervention group (Group 1). In 2015, a protocol was initiated for peri-procedural management of patients presenting in AF, new onset or previously known. All consecutive patients after initiation of the protocol from October 2015 to September 2016 were analyzed as the post intervention group (Group 2). Patients with heart failure, hypotension, or chest pain were excluded from the protocol.

**Results:**

One hundred nine and 141 patients were included in Groups 1 and Group 2, respectively. Following protocol initiation, patients were less likely to present to the ED (6.4% Group 1 vs. 1.4% Group 2, RR 0.22, *p* = 0.04). There was also a trend towards a reduction in procedure cancelations (5.5% Group 1 vs. 1.4% Group 2, RR 0.26, *p* = 0.08). All attempted procedures were completed and there were no complications in the intervention group.

**Conclusions:**

Implementation of a standardized protocol for management of atrial fibrillation in patients presenting for outpatient gastrointestinal endoscopic procedures resulted in a significant decrease in emergency department visits with an additional trend toward decreased procedural cancellations without an increased risk of complications.

## Background

Atrial fibrillation (AF) is one of  the most common cardiac arrhythmias, impacting approximately 2.7 to 6 million people in the United States (U.S.) and the prevalence is increasing [1]. An estimated 6 billion dollars is spent annually in the U.S. as a result of AF with inpatient admissions and emergency departments (ED) visits, accounting for the bulk of expenses [2, 3]. Several studies have demonstrated that protocol-driven ED management of AF may decrease admissions, length of stay and costs [4–10]. However, there is a paucity of evidence with respect to the usefulness of AF management protocols in non-acute care or procedural settings. The discovery of AF by non-Cardiology providers may result in unnecessary procedure cancellations and ED referrals. Approaches to safely manage these patients during such procedures may not only improve quality of patient care but also reduce AF related costs.

At our institution, we initiated a protocol to manage patients with AF presenting for outpatient gastrointestinal (GI) endoscopic procedures. The aim of the protocol was to effectively rate control patients peri-procedurally in order to allow for procedure completion. Once the procedure was complete, communication and follow up with the patient’s primary physician was established to avoid ED referrals.

## Methods

**Fig. 1:**
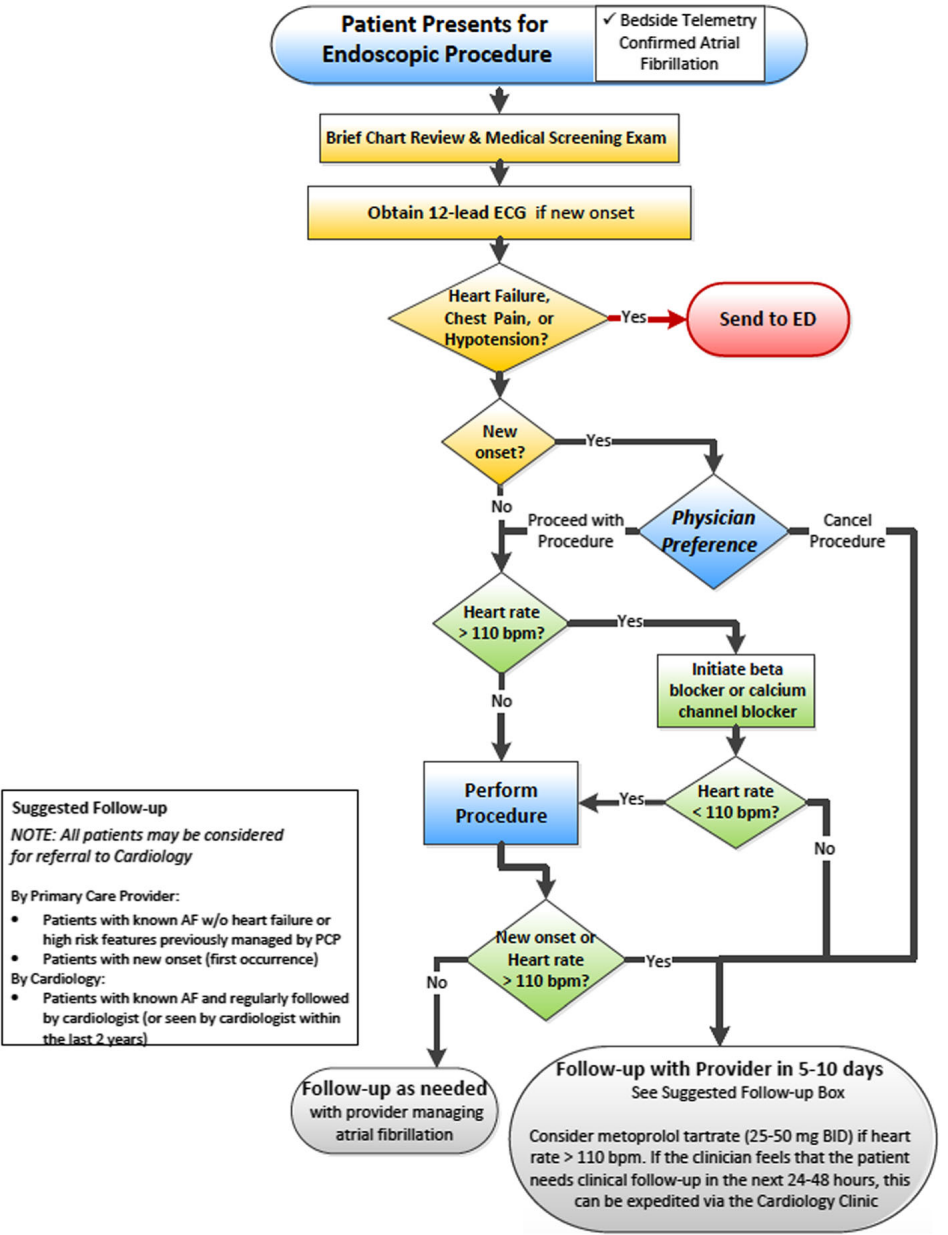
Digestive health center endoscopy atrial fibrillation management algorithm

All consecutive outpatients presenting with AF at the time of upper or lower endoscopies at the University of Wisconsin Digestive Health Center were included in the study. Patients presenting between October 2013 and September 2014 were defined as the pre-intervention group. A protocol for peri-procedural management of patients in AF was implemented in August of 2015 (Fig. 1). Patients presenting between October 2015 and September 2016 were defined as the intervention group. Patients with both new onset and known AF were included. Patients were excluded if they exhibited clinical heart failure symptoms, hypotension, or chest pain at the time of index GI procedure. The primary outcome was rate of ED referral or procedural cancellation. Secondary outcome was procedural complications. The follow-up period was 30 days after the scheduled index GI procedure.

All data are reported as mean ± SD for continuous variables and frequencies for categorical data. Continuous variables were compared by unpaired or paired two-tail t-test as appropriate. Categorical variables were compared using χ tests. The Fisher exact test was used to compare proportions. Statistical analyses were performed using R version 3.3.3 (2017-03-06). A *p*-value of < 0.05 was considered to be significant. In accordance with federal regulations, this project did not constitute research as defined under 45 CFR 46.102(d). Therefore, this project did not require IRB review.

## Results

### Study population

One-hundred nine patients were included in the pre-intervention group (Group 1) and 141 were included in the intervention group (Group 2). The overall incidence of AF in patients presenting for outpatient endoscopy was approximately 1%. No patients were excluded from the study and 30-day follow-up data was available on all patients. Analysis of baseline demographics did not show any statistically significant differences between the two groups (Table 1).

**Table 1:** Baseline demographics

Characteristics	Group 1 (Pre-Intervention)	Group 2 (Intervention)	*p*-value
	(*n* = 109)	(*n* = 141)	
Age, years	70 ± 9	72 ± 9	0.25
Female	32 (29%)	37 (26%)	0.586
Hypertension	80 (73%)	109 (77%)	0.48
Diabetes	30 (28%)	43 (31%)	0.61
EF < 40%	6 (6%)	6 (4%)	0.648
History of stroke	19 (17%)	16 (11%)	0.17
CAD/PVD	25 (23%)	38 (27%)	0.47
New AF Diagnosis, n (%)	8 (7%)	15 (11%)	0.37
Mean CHA_2_DS_2_VASc in all patients	2.99	3.05	0.78
Mean CHA_2_DS_2_VASc in new AF patients	2.00	3.27	0.09
Average SBP	128	129	0.53
Average DBP	72	72	0.88
Average HR	76	74	0.32

*AF* atrial fibrillation, *CAD* coronary artery disease, *CHA*_*2*_*DS*_*2*_*VASc* congestive heart failure, hypertension, age, diabetes, stroke/transient ischemic attack/thromboembolism, vascular disease, age 65–75 years, sex category, *EF* ejection fraction, *PVD* peripheral vascular disease, *HR* heart rate, *SBP* systolic blood pressure, *DBP* diastolic blood pressure. Values are mean ± standard deviation for continuous data and n (%) for categorical data

### Patient outcomes

Following protocol implementation, there was a statistically significant reduction in ED referrals for patients presenting with AF at the time of the GI procedure (6.4% Group 1 vs. 1.4% Group 2; *p* = 0.04). Additionally, there was a trend towards reduced procedural cancellations (5.5% Group 1 vs. 1.4% in Group 2; *p* = 0.08). There was also a trend towards more frequent administration of AV nodal blocking agents after algorithm implementation (0.9% Group 1 vs. 2.8% Group 2; *p* = 0.28). There was no significant difference in incidence of new AF between groups (7% in Group 1 vs 11% in Group 2; *p* = 0.37). Five patients in Group 1 developed complications during the procedure including bradycardia in absence of additional nodal blocking agents (2), bowel perforation (1), g-tube malfunction (1), and hypoglycemia (1). No patients in Group 2 developed complications (Table 2).

**Table 2:** Patient outcomes

Outcome	Pre-Intervention	Intervention	*p*-value
	(*n* = 109)	(*n* = 141)	
ED Referral	7 (6.4%)	2 (1.4%)	0.04
ED Referrals Admitted	3 (43%)	2 (100%)	0.19
Average LOS if Admitted (days)	2	1.5	0.51
Procedural Cancellations	6 (5.5%)	2 (1.4%)	0.08
Procedural Complications	5 (4.5%)	0	0.01
Beta Blocker/CCB given	1 (0.9%)	4 (2.8%)	0.28
Stroke or Thromboembolic events within 30 days	0	0	n/a

*CCB* calcium channel blockers, *ED* emergency department, *LOS* Length of Stay. Values are mean ± standard deviation for continuous data and n (%) for categorical data

### Post-procedure follow-up

All patients with new AF in Group 1 were seen by a provider within 30 days of the procedure vs. 73.3% of patients in Group 2. For those patients who were referred to the ED, 43% in Group 1 were admitted to the hospital versus all patients in Group 2. There was no difference in length of hospital stay between the two groups. There was no difference in the percentage of patients with CHA_2_DS_2_VASc ≥ 2 between Group 1 and Group 2 (84.4% vs. 83.7%; *p* = 0.89). There was no significant difference in anticoagulation rates in patients with a CHA_2_DS_2_VASc ≥ 2 between the groups (71.7% Group 1 vs. 76.9% Group 2; *p* = 0.20). For patients with newly diagnosed AF at the time of their procedure, there was no significant difference in anticoagulation rates in patients with a CHA_2_DS_2_VASc ≥ 2 between the two groups 30 days post procedure (66.7% Group 1 vs. 50.0% Group 1; p-0.53 (Table 3)).

**Table 3:** Patient anticoagulation status

	Pre-intervention	Intervention	*p*-value
Patients CHA_2_DS_2_VASc ≥ 2	92 (84.4%)	118 (83.7%)	0.89
Patients with CHA_2_DS_2_VASc ≥ 2 on anticoagulation	66 (71.7%)	90 (76.3%)	0.20
Newly diagnosed AF and CHADS_2_VASc ≥ 2	6 (75.0%)	12 (80.0%)	0.79
Newly diagnosed AF and CHA_2_DS_2_VASc ≥ 2 on anticoagulation	4 (66.7%)	6 (50.0%)	0.53

*CHA*_*2*_*DS*_*2*_*VASc* congestive heart failure, hypertension, age, diabetes, stroke/transient ischemic attack/thromboembolism, vascular disease, age 65–75 years, sex category. Values are mean ± standard deviation for continuous data and n (%) for categorical data

## Discussion

The present study supports the safety and efficacy of a protocol to manage AF in the outpatient GI endoscopy setting. Not only was there a significant decrease in the number of patients referred to the ED following algorithm implantation, but there was also a trend towards decreased procedural cancellations without an increase in adverse events.

Many patients presenting with asymptomatic or minimally symptomatic AF can be safely managed in the outpatient setting. However, the comfort level may be lower for management of AF by non-cardiology subspecialties and therefore can result in unnecessary referral to the ED or urgent care facilities as well as cancellation of procedures. Armed with a protocol developed in collaboration with cardiovascular specialists, GI providers had increased comfort levels to continue the planned procedures and then arrange for outpatient follow-up for those patients presenting in AF. As shown in the analysis, a sizeable percentage of the AF patients were newly diagnosed at the time of their GI procedure. There has been an increased awareness of the substantial incidence of subclinical AF in older patients through systematic screening efforts [11, 12]. Patients presenting for outpatient GI procedures often have continuous telemetry, pulse oximetry and vitals that lead to the recognition of AF in patients otherwise not undergoing screening and therefore serves as a potential source of newly diagnosed AF. As our study demonstrates, there is a significant number of patients presenting for endoscopies with previously undiagnosed AF. This study provides data to support performing GI endoscopies for patients with newly diagnosed asymptomatic or minimally symptomatic AF. Moreover, patients undergoing outpatient colonoscopies typically present after undergoing a bowel preparation and thus cancellations of these procedures can be burdensome for patients.

Formalized protocols have the ability to standardize care and reduce unnecessary healthcare utilization. In the ED setting, several studies have shown that use of AF management protocols are successful in reducing hospital admissions without increasing adverse events [4, 5, 7, 9, 10, 13]. The present study is the first of its kind to support the report the implementation of an algorithm for management of patients with AF in a setting outside of the ED. The outpatient GI setting was chosen owing to the inherent low procedural risks and short procedural duration. Protocols such as the one used in this study could be applied to other low risk outpatient arenas to improve quality of care for AF patients.

There are several limitations of our study. First, we used a retrospective control group which carries the potential for diagnostic and treatment differences. However, it is unlikely that within the overall time period that management would significantly differ and both the nursing and physician personnel were unchanged during this study period. Second, this is a single center study with a relatively small cohort of patients. Thus, the study may be underpowered to evaluate true differences seen as trends with respect to procedural cancellations and administration of AV nodal blocking agents. Third, concern exists about spontaneous cardioversion to sinus rhythm in individuals with a CHA_2_DS_2_VASc ≥ 2 who are not on anticoagulation during a procedure and have newly diagnosed AF. AF guidelines recommend long-term anticoagulation in those patients [2, 6, 14]. While data has shown that bridging anticoagulation in peri-procedural settings is not necessary, there is a lack of data regarding risk of spontaneous cardioversion during endoscopic procedures, and future investigation of this topic may aid further understanding of this risk [15]. Notably, no complications from thromboembolic events were noted during the study period. Lastly, our study did not evaluate the potential cost savings with the associated decreased ED referrals and procedural cancelations.

To assess generalizability, this study would ideally be followed by a multicenter study with a larger cohort of patients. Additionally, changes to the protocol to help facilitate appropriate anticoagulation in patients with newly diagnosed AF could help reduce morbidity and mortality.

## Conclusions

Implementation of a novel protocol for AF management during outpatient GI procedures decreased ED referrals with a trend towards decreased procedural cancellations without any direct adverse events. Such protocols provide a practical method for non-cardiac specialists to manage patients with cardiovascular problems and reduce unnecessary healthcare utilization. This is the first study to support use of an algorithm to manage patients with AF in low risk ambulatory procedural settings.

## Abbreviations

AF: Atrial Fibrillation
CAD: Coronary Artery Disease
DBP: Diastolic Blood pressure
ED: Emergency Department
EF: Ejection Fraction
GI: Gastrointestinal
HR: Heart Rate
HTN: Hypertension
SBP: Systolic Blood Pressure
U.S.: United States
